# *Staphylococcus aureus* wraps around *Candida albicans* and synergistically escapes from Neutrophil extracellular traps

**DOI:** 10.3389/fimmu.2024.1422440

**Published:** 2024-07-10

**Authors:** Qi Jing, Rui Liu, Qingsong Jiang, Yingshuang Liu, Jinzhi He, Xuedong Zhou, Ollie Yiru Yu, Chun-Hung Chu, Lei Cheng, Biao Ren, Mingyun Li

**Affiliations:** ^1^ State Key Laboratory of Oral Diseases, National Clinical Research Center for Oral Diseases, West China School of Stomatology, Sichuan University, Chengdu, China; ^2^ Department of Orthodontics, West China School of Stomatology, Sichuan University, Chengdu, China; ^3^ Department of Operative Dentistry and Endodontics, West China Hospital of Stomatology, Sichuan University, Chengdu, China; ^4^ Faculty of Dentistry, the University of Hong Kong, Hong Kong, Hong Kong SAR, China

**Keywords:** *Candida albicans*, *Staphylococcus aureus*, subcutaneous infection, neutrophil extracellular traps (NETs), co-infection, immune response

## Abstract

**Background:**

NETs, a unique neutrophil immune mechanism, are vital in defending against microbial invasions. Understanding the mechanisms of co-infection by *Candida albicans* and *Staphylococcus aureus*, which often leads to higher mortality and poorer prognosis, is crucial for studying infection progression.

**Methods:**

In our study, we established a mouse model of subcutaneous infection to characterize the inflammation induced by co-infection. By purifying and extracting NETs to interact with microorganisms, we delve into the differences in their interactions with various microbial species. Additionally, we investigated the differences in NETs production by neutrophils in response to single or mixed microorganisms through the interaction between neutrophils and these microorganisms. Furthermore, we analyzed the gene expression differences during co-infection using transcriptomics.

**Results:**

In vivo, *C. albicans* infections tend to aggregate, while *S. aureus* infections are more diffuse. In cases of co-infection, *S. aureus* adheres to and wraps *C. albicans*. NETs exhibit strong killing capability against *C. albicans* but weaker efficacy against *S. aureus*. When NETs interact with mixed microorganisms, they preferentially target and kill the outer layer of *S. aureus*. In the early stages, neutrophils primarily rely on phagocytosis to kill *S. aureus*, but as the bacteria accumulate, they stimulate neutrophils to produce NETs. Interestingly, in the presence of neutrophils, *S. aureus* promotes the proliferation and hyphal growth of *C. albicans*.

**Conclusion:**

Our research has showed substantial differences in the progression of co-infections compared to single-microbial infections, thereby providing scientific evidence for NETs as potential therapeutic targets in the treatment of co-infections.

## 1 Introduction

The occurrence of infectious inflammation involves numerous complex pathogenic factors, with the disruption of microbial balance and immune homeostasis as the primary contributors (1) Polymorphonuclear Leukocytes (PMNs), often referred to as neutrophils, constitute the first line of defense against pathogen infections (2). Imbalance in the interaction between PMNs and microorganisms can lead to disease manifestations (3). Upon stimulation by pathogenic microorganisms or chemical substances, neutrophils release discrete DNA that combines with cytoplasmic granule proteins to form a fibrous network structure known as NETs. NETs play a pivotal role in the innate immune response mediated by neutrophils (4). Their unique network structure enables NETs to capture pathogens and restrict their dissemination, while the antimicrobial components they contain exert killing effects on the pathogens. The functional role of NETs in the development of diseases is a forefront research topic (5, 6), and their mechanisms in inflammation-related diseases (7–9) have been extensively studied. Current research primarily focuses on the interplay between a single microbe and NETs, yet coinfection with multiple microorganisms is a significant factor that aggravates infections. Co-detection rates of *C. albicans* and *S. aureus* in infectious diseases are high, and their associated mortality rates are similarly elevated (10–14). However, the co-interaction mechanisms between these two microorganisms with NETs remain unexplored.

The formation of NETs occurs through two main mechanisms. One involves nuclear depolarization, dissolution of the nuclear membrane, decondensation of chromatin, and then rupture of the plasma membrane to release the NETs. Alternatively, neutrophils can release nuclear chromatin and granule proteins while maintaining their non-nucleated state, allowing them to continue their phagocytic functions (15). Moreover, depending on the type of chemical stimulus or pathogen, NETs can be formed through different mechanisms, adding complexity and posing a challenge to the study of NETs (16).

*S. aureus* induces the production of NETs through the secretion of proteins such as Staphylococcal Protein A (SpA) and Leukocidin GH (Luk GH) (17–19). To evade the lethal effects of NETs, it employs nucleases to degrade the DNA structure of NETs and utilizes Extracellular Adherence Protein (Eap) to bind to DNA, inhibiting the formation of NETs (20). Interestingly, it has been observed that *S. aureus* can induce excessive formation of NETs, leading to the destruction of host tissues, facilitation of bacterial dissemination, and maintenance of chronic infections (21).

*C. albicans*, a dimorphic fungus, primarily exhibits its pathogenicity in the hyphal phase. The yeast form of *C. albicans* primarily relies on phagocytosis for elimination, whereas the larger hyphal form employs the release of NETs for eradication (22, 23). Neutrophils selectively release NETs based on their perception of the microorganism’s size, a process facilitated by Neutrophil Elastase (NE) (24). Although *C. albicans* secretes nucleases, current research indicates that DNA degradation alone cannot fully protect the fungus from the effects of various antifungal proteins present in NETs (25).

The aforementioned studies have demonstrated that both *C. albicans* and *S. aureus* possess the ability to induce NETs production as well as evade NETs. However, in the context of co-infection, these microorganisms engage in interspecies interactions, resulting in the formation of mixed biofilms. Research has shown that *S. aureus* adheres to the hyphae of *C. albicans* through the agglutinin-like sequence 3 (Als3), and the fibrin formed by *S. aureus* around *C. albicans* enables the latter to evade the killing effect of immune cells (26, 27). Moreover, and the virulence factor Eap produced by *S. aureus* inhibits hypha-induced NETs release (23, 28). Nevertheless, the role of NETs in this process has not been investigated.

Neutrophils are considered short-lived effector cells that undergo apoptosis in damaged tissues during infectious inflammation. *In vitro*, the lifespan of mouse neutrophils is approximately 24 h, but their survival is prolonged in inflammatory tissues (29). When neutrophils enter peripheral tissues, their phenotype undergoes further adjustments. Therefore, during migration and function, although their residence time in tissues is limited, they can adapt their characteristics to support immune homeostasis in organs, remaining in the inflamed tissue for over 24 h (30) and playing a pivotal role.

Typically, animal infection models related to *C. albicans* primarily involve *in situ* models of oral mucosa (31, 32) and intestinal mucosa (33), where colonization often necessitates the creation of a susceptible environment with reduced immunity. This is often achieved through the administration of immunosuppressive drugs (34) or by controlling the age of animal models. Taking into account various interfering factors during colonization, such as the impact of swallowing or intestinal peristalsis on microbial colonization in the oral cavity (35, 36) or intestinal mucosa (33), as well as the effect of immunosuppressive drugs on neutrophil immunity, we ultimately opted for subcutaneous infection to establish a mouse co-infection model. The utilization of subcutaneous injection for establishing infection models with a single microorganism is a common and well-established approach (37, 38), analogous to modeling cancer cells *in situ* (39) or ectopically (40, 41), which involves injecting the microorganisms into a specific location to achieve the desired modeling purpose.

This study found that in the case of co-infection, *S. aureus* not only significantly stimulates the proliferation and hyphal development of *C. albicans* but also envelops it within its distinctive structure. This encapsulation effectively impedes the phagocytic capabilities of neutrophils and triggers the rapid deployment of NETs. Notably, when confronted with this mixed microbiota, NETs primarily target the outer layer of *S. aureus*, exhibiting a comparatively limited killing effect on the bacterium. Consequently, this attribute inadvertently aids the *C. albicans* wrapped by *S. aureus* to evade NETs-mediated killing, thereby enhancing its survival rate. This collaborative evasion mechanism between *S. aureus* and *C. albicans* significantly bolsters their co-infection invasiveness, enabling both pathogens to more effectively counter the host’s immune defense system.

## 2 Materials and methods

### 2.1 Mouse experiments

The establishment of the animal model was based on a detailed protocol outlined in a previous relevant study (42). *C. albicans* sc5314 and *S. aureus* NCTC-8325-4 were cultured overnight and resuspended in 9% sterile saline. The mice(male, C57BL/6 (43), 6-8 weeks old) were divided into four groups, with at least six mice per group (Total = 24): *S. aureus* group (1 × 10^7^ CFUs in total), *C. albicans* group (1 × 10^6^ CFUs in total), the co-infection group (*S. aureus* 1 × 10^7^ CFUs + *C. albicans* 1 × 10^6^ CFUs in total), and the uninfected control group. The injection volume was 2 mL, administered via subcutaneous injection, with the concentration of strains adjusted according to the total injection volume. Mice were sacrificed at 5, 24, and 48 h post-infection (two mice per group per time point, n=2), and tissue samples were collected for histopathological and microbial load assessments. Uninfected mice served as the control group for comparative analysis. The entire experimental procedure adhered to the internationally recognized Animal Research: Reporting of *In Vivo* Experiments (ARRIVE) guidelines.

### 2.2 Neutrophil isolation

The isolation of neutrophils was carried out following the protocol established by Yansen Xiao (44), with several modifications to optimize the process. To isolate neutrophil from bone marrow, bone marrow cells from 6 to 8-weeks-old C57BL/6 mice were harvested in sterile Hank’s buffered salt solution (HBSS) without Ca^2+^/Mg^2+^ (Solarbio, China). The cell suspension was gently layered on top of a density gradient consisting of Ficoll Plus 1.077 (Solarbio, China) and 11191#RNBK6705 (Sigma, Germany), followed by centrifugation at 1,000 g for 30 min at 25°C. Unless noted otherwise, neutrophils were cultured in RPMI 1640 medium containing 10% FBS.

### 2.3 Purification of NETs

We isolated NETs from primary neutrophils of bone marrow using a previously described method with slight modifications (45, 46). Neutrophils were treated with 500 nM PMA (APExBIO, USA) (47) for 4 h. After removal of the supernatant, NETs adhered at the bottom were washed down by pipetting 2 mL of cold PBS and were centrifuged at 1,000 g at 4°C for 10 min. The cell-free supernatant containing NETs (DNA–protein complex) was collected. The DNA concentration of NETs was measured by spectrophotometry and the NETs were used for further experiments.

### 2.4 *In vitro* NET analysis

To assess NETs formation, neutrophils (1×10^5^ cells (48)) were seeded on coverslips coated with poly-L-lysine (Sigma, Germany) in 24-well plates for 30 min before adding *S. aureus* (MOI=30, MOI=100), *C. albican* (MOI=10 (49)) with *S. aureus* (MOI=100). After 4 h (50) at 37°C, neutrophils were fixed with 4% paraformaldehyde (PFA, Biosharp, China) for 10 min, washed three with PBS and permeabilized in 0.1% Triton X-100 for 5 min. Cells were blocked in PBS containing 5% BSA for 30 min, then incubated with anti-histone H3 (51) (1:200, 17168-1-AP, Proteintech, USA) and anti-MPO (1:200, 66177-1-Ig, Proteintech, USA) in blocking buffer overnight at 4°C. After three washes in PBS, cells were incubated with fluorochrome-conjugated secondary antibodies (1:500, ab97014, ab150077, Abcam, UK) for 1 h, and then counterstained with DAPI (Solarbio, China) before mounting Observation and photographing were performed with the confocal microscopy, and image processing and analysis. The procedures and methods were referenced from prior research (44).

### 2.5 Scanning Electron Microscopy

The fabrication process for SEM specimens was derived from preceding research (52). Primary neutrophils were also incubated in 24-well plates with *C. albicans*, *S. aureus* or mixed-species at specified MOIs. After gentle rinsing, electron microscope fixative was added and left for 2-16 h at 4°C. After fixation, the samples were dehydrated with a graded ethanol series (25–100%) and washed with 100% ethanol before critical point drying. Finally, they were observed using scanning electron microscopy.

### 2.6 Non-contact induction of neutrophils

Referring to previous studies (53), using a Transwell cell culture plate (0.4 µm) as a barrier, a system was established for non-contact induction of neutrophils by *S. aureus*, where the cells were placed in the upper chamber and the microorganism in the lower chamber. In each chamber, 1×10^5^ cells were inoculated, and *S. aureus* was added at a multiplicity of infection (MOI) of 100. After 4 h of co-culture, the upper chamber cells were collected and mounted on a confocal dish. The immunofluorescence staining method was consistent with the above, and the samples were observed under a confocal microscope.

### 2.7 Antimicrobial analysis

*C. albicans* and *S. aureus* were collected as described above. NETs were pre-incubated at 37°C, and diluted with sterile PBS. Gradient-diluted NETs were dispensed into 96-well plates, and *C. albicans* (1×10³ CFUs (54, 55)) or *S. aureus* (1×10^6^ CFUs (56)) were added, separately or combined. After incubation at 37°C for 2, 4, and 8 h, samples were collected, diluted, plated, and CFU counts were performed. *C. albicans* cultures were further incubated at 37°C for 24 h, while *S. aureus* cultures were incubated at 35°C for 24 h, before CFU enumeration.

### 2.8 cDNA library preparation and sequencing

The MOI of interaction between microorganisms and neutrophils was selected according to the above experimental results. *C. albicans* MOI was 10 and *S. aureus* MOI was 100. Each chamber had 1×10^5^ cells. After a co-incubation period of 4 h, the supernatant was discarded, and the remaining samples were promptly frozen in liquid nitrogen. Total RNAs was extracted for transcriptome analysis. RNA quality was determined by examining A260/A280 with Nanodrop™ OneCspectrophotometer (Thermo Fisher Scientific Inc). An amount of 2 μg of total RNAs was used for stranded RNA sequencing library preparation using KC-Digital™ Stranded mRNA Library Prep Kit for Illumina^®^ (Catalog NO. DR08502, Wuhan Seqhealth technology Co., Ltd., Wuhan, China). The kit eliminates the duplication bias during PCR and sequencing steps by using a UMI of 8 random bases to label the pre-amplified cDNA molecules. The kit eliminates duplication bias in PCR and sequencing steps, by using unique molecular identifier (UMI) of 8 random bases to label the pre-amplified cDNA molecules (57–59).

### 2.9 RNA-Seq data analysis

Raw sequencing data was first filtered by Trimmomatic (version 0.36), low-quality reads were discarded and the reads contaminated with adaptor sequences were trimmed. Deduplicated Reads were mapped to the reference genome of *C. albicans* (ASM18296v3) and *S. aureus* (ASM1342v1) using STRA software (version 2.5.3a) with default parameters. Reads mapped to the exon regions of each gene were counted by featureCounts (Subread-1.5.1; Bioconductor) and then RPKM was calculated. RPKM (Reads per Kilobase per Million Reads) was used as A measure of gene expression (60).

### 2.10 Statistical analysis

Experimental data are expressed as mean ± SD. When the experimental data were normally distributed, *t* test or one-way analysis of variance was been used. Otherwise, Kruskall-Wallis analysis was been used. Differences were considered statistically significant when *P* < 0.05. The statistical analysis of the experimental data obtained was performed using GraphPad Prism 7.

## 3 Results

### 3.1 Co-infection with *C. albicans* and *S. aureus* aggravated the infection

**Figure 1:**
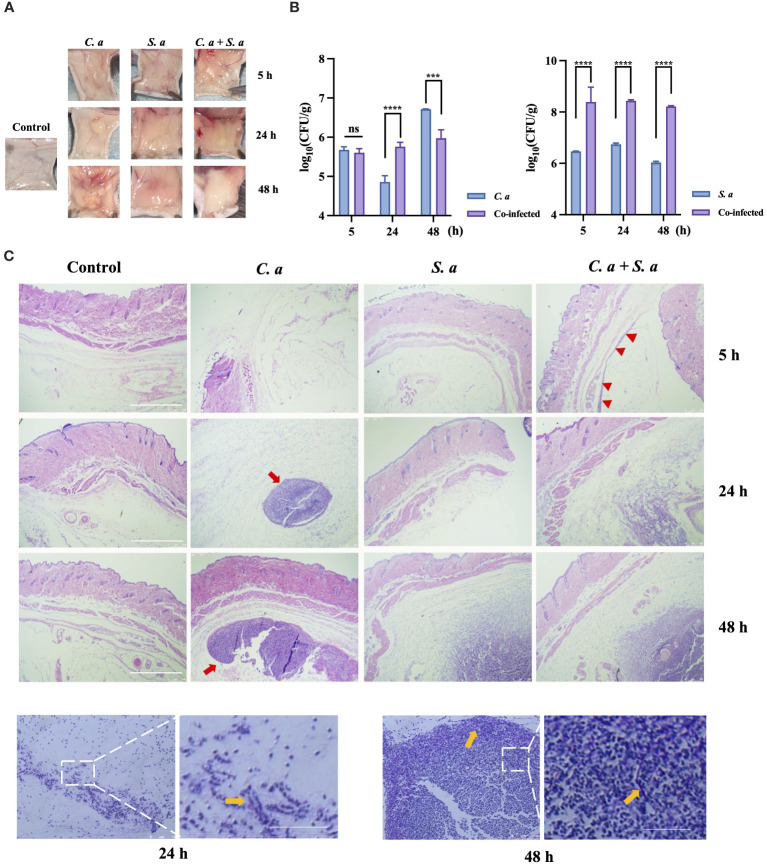
Co-infection of *C. albicans* and *S. aureus* leads to increased infection. **(A)** Formation of infected masses in mice 5, 24, and 48 h after subcutaneous injection. **(B)** Microbial colony counts of subcutaneous infected masses. *** *P* < 0.001, **** *P* < 0.0001, ns: no significance. **(C)** Representative microphotographs of pathological changes at indicated times in subcutaneous infected mass. Scale bars, 750 μm **(D)** PAS staining of *C. albicans* infected mass at 24 h, 48 h. Scale bars, 200 μm. *S. a*: *S. aureus*; *C. a*: *C albicans*; Red arrow: infected area; Yellow arrow: *C. albicans* hyphae.

To compare the characteristics of single-infections and co-infections *in vivo*, we have established a mouse subcutaneous infection model (**Figure 1**). Our findings showed (**Figure 1A**) that when *C. albicans* infects alone, localized aggregations of infection appear within 5 h, developing into spherical or ovoid, yellowish, purulent lesions that progressively enlarge a period of 24 to 48 h. In contrast, *S. aureus* infection manifests as diffuse subcutaneous thickening accompanied with a yellowish hue. In the co-infection group, comprising *C. albicans* and *S. aureus*, localized subcutaneous thickening centered around a yellowish aggregate is observed at 24 hours. After 48 h, the color fades while the thickness intensifies.

To illuminate the effects of co-infection on the proliferative potential of these microorganisms, we conducted microbial load counting on the infected tissues (**Figure 1B**). Our results indicate that the tissue load of *C. albicans* is higher in the co-infection group compared to the single-microbial infection at 24 h. Similarly, the tissue load of *S. aureus* is also elevated in the co-infection group, as compared to the single-microbial infection, at 5, 24, and 48 h.

HE staining (**Figure 1C**) showed that *C. albicans* infection exhibits a progressively expanding aggregation pattern over time, whereas *S. aureus* infection demonstrates a diffuse pattern in the infected area specifically at 48 h. Intriguingly, in the co-infection group, an infection band is noticeable at 5 h, which intensifies over time, ultimately culminating in a two-layered structure at 48 h: an outer diffuse layer, surrounding an inner aggregated layer.

To further investigate the characteristics of *C. albicans* to resist neutrophil-mediated immunity *In vivo*, we performed Periodic Acid-Schiff (PAS) staining on *C. albicans*-infected tissues at 24 and 48 h (**Figure 1D**). The staining showed the presence of hyphae of *C. albicans* at the edges of the infected sites.

### 3.2 Killing ability of NETs against *C. albicans* and *S. aureus*

**Figure 2:**
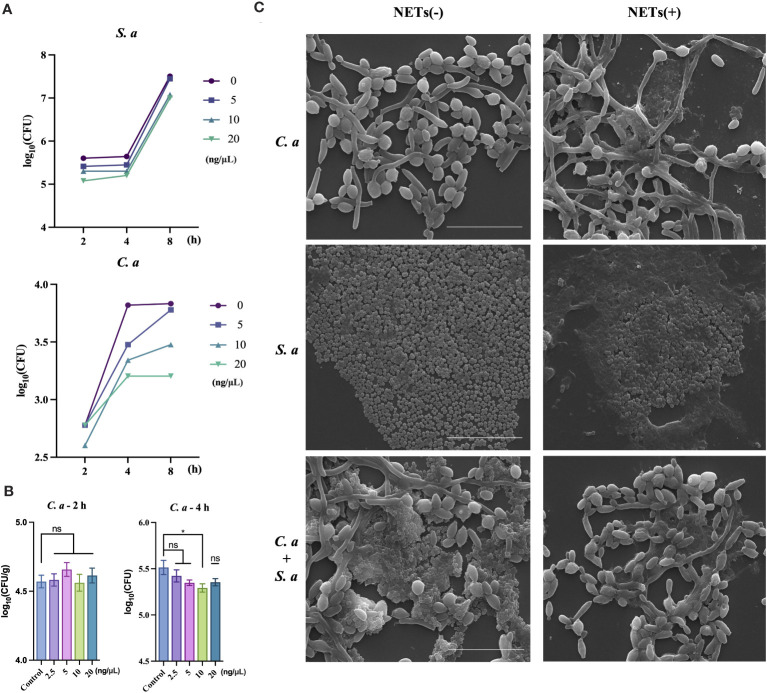
*C. albicans* growth inhibited by NETs and protected by *S. aureus* during co-infection. **(A)** Colony counts of *C. albicans* and *S. aureus* assessed at 2, 4, and 8 h post-treatment with NETs at 20, 10, and 5 ng/μL. **(B)** Colony counts of *C. albicans* interacting with NETs evaluated at 2.5, 5, 10, and 20 ng/μL after 2 and 4 h incubation. * *P* < 0.05. **(C)** SEM visualization of *C. albicans* and *S. aureus* individually or combined with NETs (20 ng/μL) for 4 h. Scale bars, 20 μm.

After conducting initial *in vivo* experiments aimed at examining the co-infection characteristics of two microorganisms, we intended to further investigate whether these identified characteristics are associated with NETs. To accomplish this, we conducted a purified extraction of NETs *in vitro* and exposed them to both single-species and mixed-species cultures of the microorganisms. This approach was designed to characterize the direct effects of NETs on the microorganisms. Under resource constraints, we utilized varying concentration gradients to evaluate the antibacterial efficacy of NETs against *C. albicans* and *S. aureus* (**Figures 2A, B**). At the peak concentration of 20 ng/μL (**Figure 2A**), both microbial species experienced pronounced inhibition of colony growth, with *C. albicans* exhibiting notably higher sensitivity compared to *S. aureus* at the 4 h interval. In a further exploration of NETs’ ability to inhibit the growth of *C. albicans* (**Figure 2B**), we observed no substantial changes in the number of *C. albicans* cells after 2 h of NETs exposure. However, after 4 h of treatment, a distinct difference in the number of *C. albicans* cells compared to the control group was observed.

Following the quantification of NETs’ differential killing effects on the two microorganisms, we further examined the morphological changes in the microorganisms after NETs treatment using SEM. SEM analysis showed (**Figure 2C**) that when NETs were applied solely to *C. albicans*, fungal hyphae were still observed to elongate notably, with NETs adhering to the surface of the hyphae. However, in the case of co-infections, there was a pronounced reduction in the number of *S. aureus* cells on the outer layer, while the hyphae of *C. albicans* did not exhibit elongation compared to the blank control group.

### 3.3 *C. albicans* and *S. aureus* stimulate neutrophils to generate NETs

In our experiments investigating the interaction between NETs and microorganisms, we observed significant differences in the killing effect of NETs against two distinct microorganisms. Specifically, the killing ability of NETs against *S. aureus* caught our attention, leading us to hypothesize that neutrophils exhibit substantial differences in their specific immune responses towards these two distinct microbial entities. To further explore the immune mechanisms between neutrophils and these morphologically distinct microorganisms, we isolated primary neutrophils and examined their interactions with the microorganisms.

**Figure 3:**
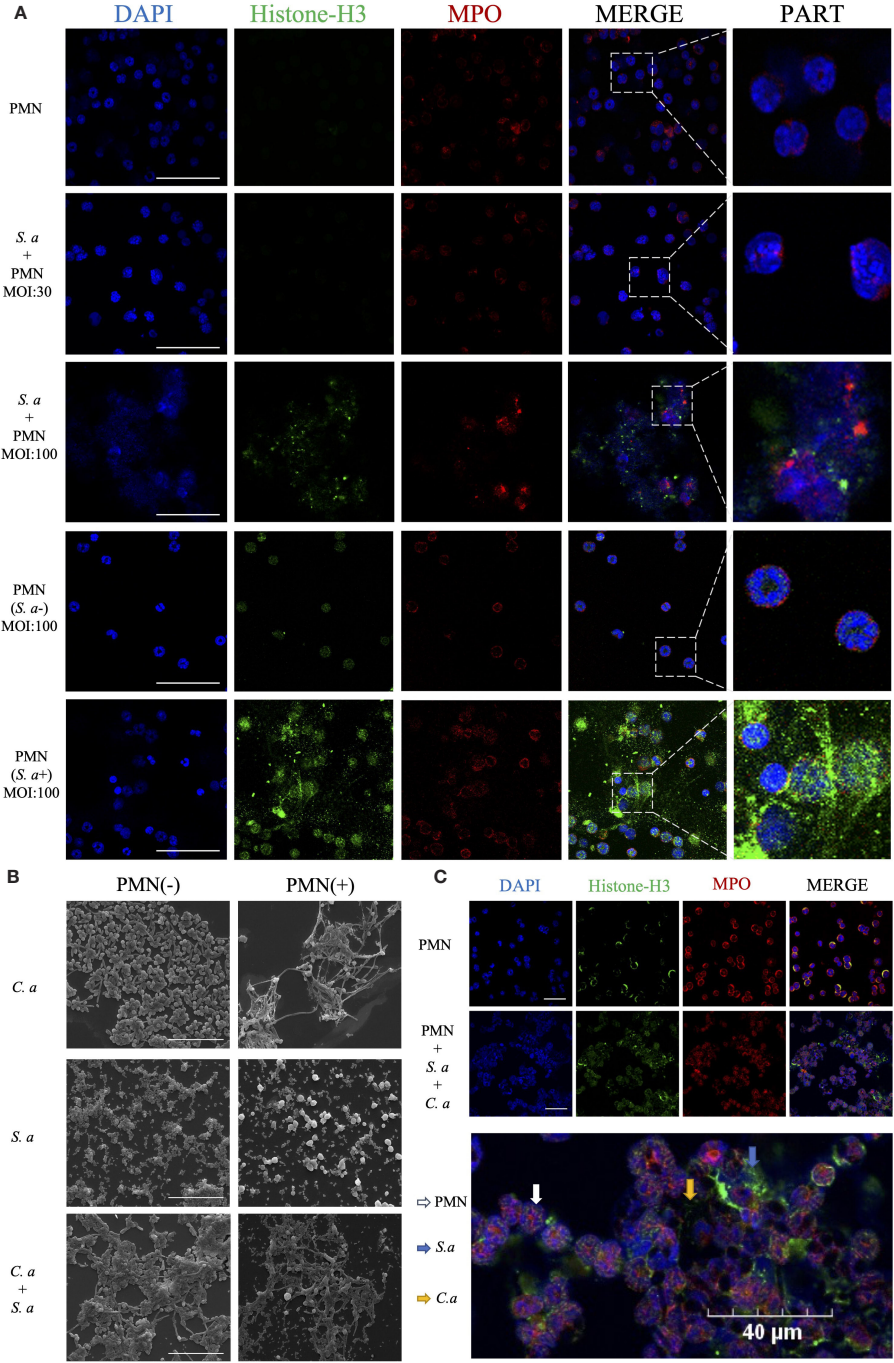
*C. albicans and S. aureus* stimulate neutrophils to generate NETs. **(A)** Visualization of the interaction between *S. aureus* and neutrophils using confocal microscopy after incubation for 4 h under different infection doses (MOI: 30, 100), and isolation of *S. aureus* (MOI: 100) with simultaneous visualization of NET production. Scale bars, 50 μm. **(B)** SEM visualization of *C. albicans* (MOI: 10) and *S. aureus* (MOI: 100) individually or in combination with neutrophils. Scale bars, 20 µm. **(C)** Confocal microscopy images showing co-localization of Histone-H3 and MPO. Neutrophils were induced for 4 h by co-stimulation with *C. albicans* (MOI: 10) and *S. aureus* (MOI: 100). Scale bars, 40 μm. The NETs were stained with Histone-H3 (green), MPO (red) and DAPI (blue).

In the co-culture group of *S. aureus* and neutrophils (**Figure 3A**), neutrophils in the low MOI group exhibited a significant number of phagocytosed *S. aureus*. However, in the high MOI group, a substantial production of NETs was detected. To investigate the role of phagocytosis in the formation of NETs induced by *S. aureus*, we utilized a transwell assay to mimic the interaction between neutrophils and *S. aureus* while preventing direct physical contact between them. Through this approach, we observed that neutrophils were still capable of inducing NETs generation even without phagocytosing *S. aureus*.

Comparing the mechanisms of NETs formation induced by *C. albicans* and *S. aureus*, scanning electron microscopy showed (**Figure 3B**) that in the *C. albicans* group, significant fungal hyphal growth was observed, and neutrophils released abundant fibrous NETs that entangled and wrapped the *C. albicans*. In contrast, in the *S. aureus* group, neutrophils released cytoplasmic NETs that wrapped *S. aureus*. In comparing the interaction mechanisms between neutrophils and single versus mixed microorganisms, SEM showed that upon the introduction of neutrophils, the number of *S. aureus* that surrounding *C. albicans* was reduced. Additionally, in the mixed infection group, neutrophils released cytoplasmic NETs, with a notable increase in the volume of some neutrophils.

To confirm the release of NETs during co-infection, we utilized co-localization fluorescence labeling of NETs’ signature proteins (**Figure 3C**). The experiment showed co-localized fluorescence expression around the microorganisms and neutrophils, with the fluorescence primarily concentrated around the outer layer of *S. aureus*, while exhibiting reduced expression around the hyphae of *C. albicans* in the interior.

### 3.4 *S. aureus* promotes the proliferation and hyphal growth of *C. albicans*

**Figure 4:**
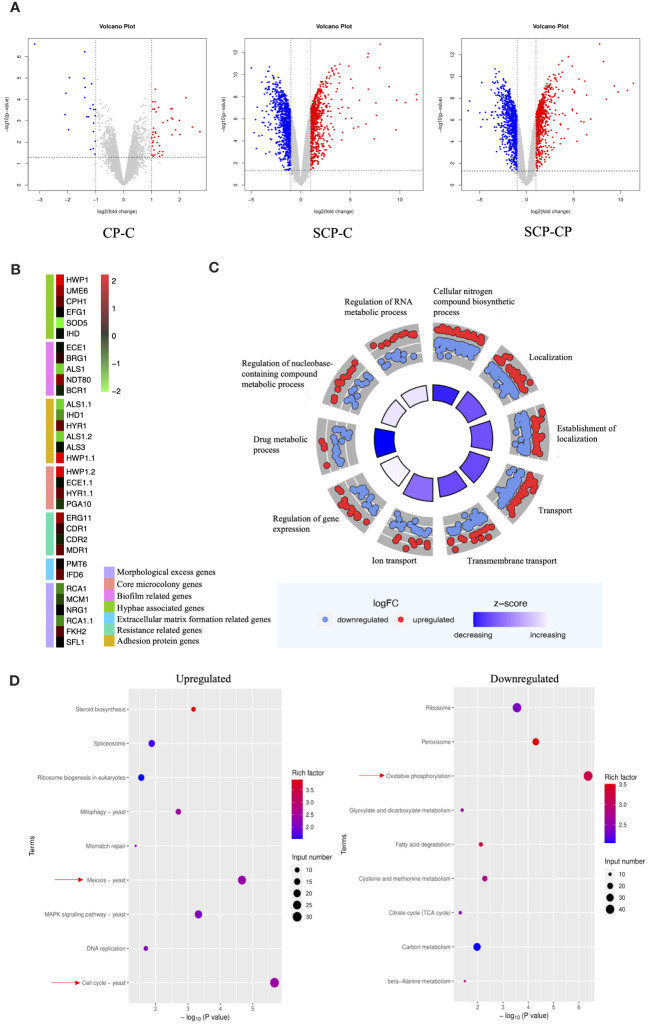
Effect of *S. aureus* on the global gene expression profile of *C. albicans*. **(A)** Volcano plot illustrating differential gene expression (DEG) patterns pre- and post-inoculation with *S. aureus*. **(B)** Selected genes differentially expressed and associated with specific mechanisms are shown in the heat map. **(C)** GO enrichment analysis of differentially expressed genes. Red or blue dots represent upregulated or downregulated genes in response to *S. aureus*, respectively. The inner cycle bars represent the statistical significance. Log_2_(FC), Log_2_(SCP group/CP group), denotes the ratio of expression levels between two samples (groups). **(D)** Enrichment analysis of KEGG pathways. Color scale represents the ratio of differentially expressed genes under the metabolic pathway to all genes annotated to the pathway, and the size of circles indicates gene numbers. X-axis, P Value. Y-axis, GO term.

To further investigate the interaction between *S. aureus* and *C. albicans* during co-infections, we employed an RNA-Seq analysis. Given the opportunistic nature of *C. albicans* as an infectious fungus, with its hyphal form being the primary pathogenic factor, we focused our attention on how this fungus behaves in the presence of *S. aureus*, particularly on its ability to resist neutrophils. To this end, we designed three experimental groups to monitor the gene expression patterns of *C. albicans* in detail. Before and after the introduction of *S. aureus*, we observed a significant increase in the number of differentially expressed genes in *C. albicans* (**Figure 4A**).Among these, the expression levels of hypha-related genes such as HWP1, UME6, CPH1, EFG1, and IHD1, as well as biofilm-related genes NDT80 and BRG1, were upregulated. While adhesin genes ALS1 and IHD1 showed downregulation, HYR1 and HWP1 exhibited upregulation. Furthermore, genes related to extracellular matrix formation (IFD6), virulence factors associated with core microcolony formation (HWP1, HYR1, SAP5, PLB1), and drug resistance (ERG11 and MDR11) were all upregulated (**Figure 4B**).

To gain a deeper understanding of these changes, we performed GO (Gene Ontology) functional enrichment analysis (**Figure 4C**) and KEGG (Kyoto Encyclopedia of Genes and Genomes) pathway enrichment analysis. GO analysis showed an upregulation of genes related to RNA metabolic processes, nucleobase-containing compound metabolic processes, and gene expression regulation, while genes involved in drug metabolic processes were downregulated. KEGG pathway analysis (**Figure 4D**) indicated that the introduction of *S. aureus* primarily upregulated genes related to meiosis and cell cycle in *C. albicans*, suggesting a potential stimulatory effect on proliferation. Conversely, genes involved in energy metabolism were significantly downregulated.

## 4 Discussion

Over the course of the study, male mice were utilized for animal modeling. Previous studies have indicated that estrogen can impact the function and recruitment of neutrophils (43, 44). Therefore, selecting male mice with stable hormone levels for immune modeling can enhance the generalizability of our experiment. The escalating invasiveness of co-infections (**Figure 1A**) highlights the growing dominance of *S. aureus* in interspecies competition (**Figure 1B**), suggesting that the immune system’s response may be influenced by microbial interactions. Closely tied to their colonization and dissemination abilities is the infective morphology of microorganisms. *S. aureus*, owing to its small, ovoid shape, exhibits a reduced tendency for colonization, facilitating the ease of infection spread (61). This is reflected in the diffuse infection patterns observed in models involving *S. aureus* (**Figure 1C**), which aligns with findings from other studies utilizing animal models injected with the bacterium (38). Conversely, *C. albicans* relies on its intricate hyphal structure for dissemination and colonization (62), leading to localized, aggregated infections.

When these two microorganisms co-infect, they exhibit a distinctive infection pattern: internal aggregation and external dissemination (**Figure 1A, C**). This pattern not only ensures the pathogens’ colonization capabilities but also promotes their further dissemination and proliferation. The encapsulation phenomenon observed *in vivo* may show a synergistic mechanism for pathogens to evade the immune system. Our *in vitro* experiments (**Figures 2C**; **3B**) further validate the existence of this encapsulation phenomenon, strengthening our hypothesis. The primary mode of invasion by *C. albicans in vivo* occurs through outward hyphal infection (**Figure 1D**), consistent with neutrophils’ rapid response to hyphae (63), which are the primary targets of immune cells *in vivo*.

Within the context of co-infections, we have delved into the functional mechanisms of neutrophil-released NETs. When evaluating the bactericidal effect of NETs against two different microorganisms, we observed contrasting results (**Figure 2A**). NETs exhibited relatively weaker killing capability against the smaller *S. aureus*. In contrast, NETs displayed robust bactericidal activity against *C. albicans*, and our experiments further analyzed the killing effect of NETs on *C. albicans*, showed significant effectiveness within 4 h (**Figure 2B**).

Intriguingly, when NETs acted solely on *C. albicans* (**Figure 2C**), they significantly induced the elongation of its hyphae. However, when NETs were exposed to a mixed population of *S. aureus* and *C. albicans*, no significant changes in the hyphal morphology of *C. albicans* were observed. This finding suggests that *S. aureus* may somehow counteract the potential effect of NETs on the hyphal growth of *C. albicans*.

Concurrently, we observed that during extraction, resuspension, isolation, or the process of cell death and lysis, NETs might lose some of their inherent properties, including cellular control and functional activity. This loss significantly impacted their morphological scaffold structure, resulting in the absence of a fibrous backbone (64, 65).

The methods of NETs production vary among different microbial species, resulting in distinct morphological variations (66). Our research showed that while both *C. albicans* and *S. aureus* can induce NETs formation, the resulting NETs exhibit significant morphological differences (**Figure 3B**). Specifically, *C. albicans*-induced NETs are fibrous and filamentous, whereas *S. aureus*-induced NETs primarily adopt a cytoplasmic morphology. This underscores the distinct pathways employed by the two microorganisms in triggering NETs production. When confronted with neutrophils, *S. aureus* is initially phagocytosed by neutrophils in large numbers, followed by the release of NETs for eradication.

Notably, during *C. albicans*’ defense against neutrophils, we did not observe phagocytosis. While numerous studies indicate that yeast-form *C. albicans* can be phagocytosed by neutrophils, in actual infection environments, the rapid release of NETs promptly stimulates the hyphal formation of *C. albicans* (22, 23). This process not only inhibits the phagocytosis of neutrophils towards the morphologically enlarged *C. albicans*, but the abundant hyphal growth also accelerates the recognition of the pathogen by neutrophils, thus promoting the continuous release of NETs.

In the process of *S. aureus* inducing NETs, it is generally believed that this occurs through a non-lethal pathway, enabling neutrophils to maintain their phagocytic capabilities after releasing NETs (66, 67). However, previous studies have not provided a clear understanding of the specific temporal relationship between these two mechanisms. To gain further insights, we conducted comparative experiments using high and low MOIs (multiplicity of infection) to observe the functional mechanisms of neutrophils at the same time points (**Figure 3A**). Our research findings indicate that neutrophils prioritize phagocytosis to clear *S. aureus* pathogens, followed by releasing NETs to target bacteria that escape phagocytosis. Notably, the direct phagocytic capacity of neutrophils against *S. aureus* is quantitatively limited. Through further analysis using a transwell system to separate *S. aureus* from neutrophils, we discovered that the production of NETs induced by *S. aureus* does not solely rely on phagocytosis as a prerequisite. Instead, certain extracellular substances released by microorganisms can trigger the release of NETs. This finding provides a novel perspective on understanding the complex interactions between neutrophils and *S. aureus*.

In our *in vitro* experiments, we observed that when two microbial species co-infect (**Figure 3B**), *S. aureus* tends to adhere to the hyphae of *C. albicans*, forming a unique biofilm structure (68, 69). This specific configuration effectively segregates *C. albicans* from direct contact with neutrophils, prompting the neutrophils to primarily target *S. aureus*. However, this adhesion phenomenon poses a challenge to the phagocytic function of neutrophils, rendering them less effective in clearing *S. aureus*.

Furthermore, due to the adherence of *S. aureus*, which leads to an increase in the size of the pathogen complex, NE within the neutrophils is rapidly activated (24), accelerating the release of NETs. Nevertheless, as *C. albicans* is partially shielded by the *S. aureus* coating, it is not fully exposed to the NETs, resulting in a reduced direct killing effect of NETs on *C. albicans*. Notably, the morphology of NETs induced by co-infection resembles that triggered solely by *S. aureus*. This observation aligns with our hypothesis that the primary target of NETs shifts from *C. albicans* to the *S. aureus*.

In a transcriptome analysis (**Figure 4**), it was discovered that *S. aureus* not only stimulated an upregulation of hypha-related genes (70, 71) in *C. albicans* but also significantly upregulated genes involved in pathways representing proliferation rates. *S. aureus* stimulated key genes related to core microcolonies formation (72) in *C. albicans* hyphae and certain biofilm-related genes (70), facilitating rapid colonization and increasing drug resistance (73). This discovery is mutually corroborated by our findings of an increased invasive capability of *C. albicans in vivo* and the production of hyphae observed *in vitro*. Moreover, during the colonization of epithelial cells by *C. albicans* hyphae, the adhesive proteins Als3 and HWP1 play pivotal roles (74, 75). Notably, in the transcriptome, we found that while Als3 expression remained largely unchanged, the expression of HWP1 was upregulated, further confirming the importance of Als3 in *C. albicans* for maintaining the stability of epithelial cell-associated adhesion complexes. The variation in virulence factors of *S. aureus* is closely associated with its infection stages. Remarkably, the matrix secreted by *S. aureus* while wrapping *C. albicans* may be a critical factor contributing to increased drug resistance (76). This enhanced resistance has the potential to adversely affect the bactericidal effect of NETs. Specifically, by stimulating the formation of hyphae in *C. albicans*, *S. aureus* not only impedes the direct phagocytosis of *C. albicans* by neutrophils but also accelerates the release of NETs, potentially promoting the progression of infection.

Based on our results, there appears to be a connection between the progression of co-infection and the effects of NETs release. We speculate that the release and killing efficiency of NETs under co-infection conditions may be influenced by various factors that are less significant in mono-infection. Therefore, further research is required to delve deeper into these differential mechanisms, particularly identifying which factors impact NETs release and function compared to mono-infection.

## 5 Conclusions

This study conducted a preliminary investigation into the progression of co-infection between *C. albicans* and *S. aureus*, as well as that of single-microbe infection, by constructing a co-infection model. Our findings showed significant differences in NETosis during co-infection compared to single-microbe infection, offering novel insights for controlling the progression of co-infection.

## Data availability statement

The data presented in the study are deposited in the NCBI Sequence Readrchive (SRA) repository, accession number: PRJNA1123423.

## Ethics statement

The animal study was approved by the policy of Sichuan University and West China School of Stomatology, and the protocol was approved by the Ethical Committee of West China School of Stomatology, Sichuan University (Chengdu, China) (Project identification code: WCHSIRB-D-2020-326, approval date: 10 September 2020). The study was conducted in accordance with the local legislation and institutional requirements.

## Author contributions

QJ: Data curation, Formal analysis, Funding acquisition, Methodology, Writing – original draft. RL: Data curation, Methodology, Validation, Writing – original draft. QsJ: Data curation, Methodology, Validation, Writing – original draft. YL: Data curation, Methodology, Validation, Writing – original draft. JH: Data curation, Formal analysis, Methodology, Writing – review & editing. XZ: Data curation, Formal analysis, Methodology, Writing – review & editing. OY: Data curation, Formal analysis, Methodology, Supervision, Writing – review & editing. C-HC: Data curation, Formal analysis, Methodology, Supervision, Writing – review & editing. LC: Investigation, Supervision, Validation, Writing – review & editing. BR: Investigation, Supervision, Validation, Writing – review & editing. ML: Conceptualization, Data curation, Formal analysis, Funding acquisition, Investigation, Methodology, Supervision, Writing – review & editing.
